# Comprehensive Improvement of Binocular Structured Light Calibration Method Based on Radical-Conservative Cooperative Particle Swarm

**DOI:** 10.3390/s24248155

**Published:** 2024-12-20

**Authors:** Jun Ma, Xing Meng, Haoseng Wang, Fangdi Jiang, Shifeng Wang, Sarath Kodagoda

**Affiliations:** 1 School of Optoelectronic Engineering, Changchun University of Science and Technology, Changchun 130022, China; mjmjmj12340@163.com (J.M.); mengxin126536@163.com (X.M.); haosenwang0813@163.com (H.W.); jiangfangdi@outlook.com (F.J.); 2 Zhongshan Institute, Changchun University of Science and Technology, Zhongshan 528400, China; 3 Faculty of Engineering and IT, University of Technology Sydney, Sydney 2052, Australia; sarath.kodagoda@uts.edu.au

**Keywords:** structured light, camera calibration, 3D reconstruction

## Abstract

To achieve high-precision 3D reconstruction, a comprehensive improvement has been made to the binocular structured light calibration method. During the calibration process, the calibration object’s imaging quality and the camera parameters’ nonlinear optimization effect directly affect the caibration accuracy. Firstly, to address the issue of poor imaging quality of the calibration object under tilted conditions, a pixel-level adaptive fill light method was designed using the programmable light intensity feature of the structured light projector, allowing the calibration object to receive uniform lighting and thus improve the quality of the captured images. Then, collaborative Particle Swarm Optimization was studied to optimize the camera parameters. Compared with other optimization algorithms, this algorithm has higher global search capability and can obtain more accurate camera parameters. Under comprehensive improvement, the 3D reconstruction accuracy of binocular structured light is 0.053 mm, showing a 36.33% improvement in reconstruction accuracy compared to mainstream calibration methods.

## 1. Introduction

Three-dimensional measurement using structured light has been widely used in reverse engineering and surface digitization due to its characteristics of non-contact, high efficiency, and fast speed [1,2]. Structured light includes point structured light, line structured light, and plane structured light. Among them, binocular plane structured light has been widely used due to its high reconstruction accuracy and efficiency. However, the high-precision reconstruction of binocular structured light relies on the high-precision calibration results of binocular cameras.

Traditional camera calibration methods include the Tsai two-step calibration method (the first step involves linear solving of external camera parameters using radial alignment constraints; the second involves nonlinear optimization of other parameters) and the Direct Linear Transformation (DLT) method. Traditional methods have a significant computational burden during calibration, and the computation accuracy depends on the manufacturing process of the calibration equipment [3]. Active vision calibration is characterized by high robustness and linear solvability, but it is unsuitable for situations where camera motion is unknown [4]. Self-calibration only requires establishing intrinsic relationships between images to complete the calibration, but it requires solving multiple nonlinear equations [5].

Zhang [6] proposed a method bridging the gap between traditional and self-calibration methods. This method involves establishing a model between a known parameter two-dimensional calibration board and captured images to solve for camera parameters. This method avoids the drawbacks of traditional methods, such as high requirements for calibration equipment and complexity of operation. Additionally, it offers higher accuracy than self-calibration methods, making it widely used in binocular structured light calibration. However, Zhang’s calibration method is affected by the quality of captured images and nonlinear optimization capabilities during the calibration process [7]. Researchers have improved the calibration process to address these issues and achieved good results [8].

Some scholars choose to perform gamma correction on distorted images to enhance the quality of captured images. Liu et al. [9] proposed a gamma correction method based on membership functions, which implements traditional adaptive computation of membership function parameters, greatly enhancing the brightness in dim areas of the image and improving dark detail. Cao et al. [10] introduced an improved adaptive gamma correction technique that enhances the contrast of bright images using a new negative image processing strategy and darkened images through gamma correction modulation using a truncated cumulative distribution function. This helps alleviate local over-enhancement and structural deformations in images. Li et al. [11] proposed a pixel-level gamma correction mapping for enhancing low-light images, combining pixel-level gamma correction with deep learning to iteratively enhance low-light input images based on the generated gamma-corrected images. These improvement methods are applied to distorted images; however, if external factors heavily influence image quality during capture, correction effects may still fall short of requirements.

Consequently, some scholars address the issue directly by adjusting exposure time to achieve optimal image quality. Param et al. [12] derived exposure time estimation as an optimization problem, selecting pixels from exposure pairs to minimize estimation errors caused by camera noise. This approach can perform well across various scenarios without requiring camera- and gain-specific noise parameters. Shi [13] proposed an exposure enhancement method that retains details in underexposed images based on an optimal weighted multiple exposure fusion mechanism, ensuring colors in the output image closely match those in high-exposure images while preserving details from low-exposure images. Meng et al. [14] proposed a method for restoring low-light images, utilizing complementary information from short- and long-exposure photos to fix the details and colors of the original scene. While these methods improve brightness and darkness details, changes in exposure time affect the entire image globally, impacting both dark and bright details simultaneously. Moreover, the methods by Shi and Meng rely on multiple exposure techniques, requiring the fusion of multiple images captured from the same angle, which is not permitted in high-precision calibration fields.

In nonlinear optimization, the Zhang calibration method and the MATLAB calibration toolbox employ traditional optimization algorithms like Levenberg–Marquardt and gradient descent methods. These traditional optimization algorithms suffer from poor convergence and are prone to converging to local optimal solutions. In recent years, nature-inspired swarm intelligence optimization algorithms have received widespread attention due to their ease of implementation, high accuracy, and fast convergence, achieving notable results. Examples include the Cuckoo Search algorithm (CS), Whale Optimization Algorithm (WOA), Particle Swarm Optimization (PSO), Genetic Algorithm (GA), and Differential Evolution Algorithm (DE). Lv et al. [15] investigated the optimization of camera parameters using an adaptive weighted and variant Particle Swarm Optimization algorithm, which quickly converges to the final result with fewer parameter settings. Wang et al. [16] utilized a variant of the Grey Wolf Algorithm to optimize camera parameters, incorporating a Grey Wolf optimization algorithm based on Levy flights and mutation mechanisms to iteratively calculate the optimal camera parameters. Li et al. [17] significantly enhanced the optimization process by integrating a Genetic Algorithm (GA) and Particle Swarm Optimization (PSO) into the Integrated GA and PSO (IGAPSO) technique. Although the studies above have combined intelligent swarm optimization algorithms with camera calibration, resulting in improved calibration accuracy, the essence lies in replacing the fitness function or feedback function in the optimization algorithm with the average reprojection error reflecting calibration accuracy. This combination fails to consider the diversity of feedback information from the reprojection error, as directly computing the average may lead to information loss.

This paper proposes a comprehensive improvement of binocular structured light based on conservative-aggressive collaborative Particle Swarm Optimization. Before adjusting exposure time, distances from each pixel on the calibration projector DMD [18] to the calibration board are calculated. The brightness of each emitting pixel is computed based on the relationship between light intensity and distance, enabling adaptive illumination compensation at the pixel level of the calibration board. This ensures uniform illumination patterns when capturing the calibration board from any angle. The camera parameters are optimized nonlinearly using the Particle Swarm Optimization (PSO) algorithm, and an improved version called the Radical-Conservative Cooperative Particle Swarm Optimization (RCCPSO) algorithm was proposed. In RCCPSO, particles representing the camera parameters are divided into radical and conservative categories based on their average reprojection error and reprojection direction. Radical particles focus on self-learning and maintaining population diversity to achieve high-level global exploration. Radical particles encourage the gradual convergence of conservative particles to facilitate local development. By leveraging feedback information generated during calibration, RCCPSO exhibits strong global search capabilities and can obtain more accurate calibration parameters.

This paper is divided into seven sections. The second section introduces the stereo-structured light calibration model. The principles of pixel-level illumination compensation and RCCPSO are described in the third and fourth sections. The feasibility of the proposed method is demonstrated in the fifth section of the calibration experiment, while the 3D reconstruction experiment shows that the proposed method can achieve higher reconstruction accuracy. Section six discusses the proposed method. Section seven presents the research conclusions of this paper. The code is available at https://github.com/Majun12343/RCCPSO.git (accessed on 17 December 2024).

## 2. Binocular Structured Light Calibration Model

**Figure 1:**
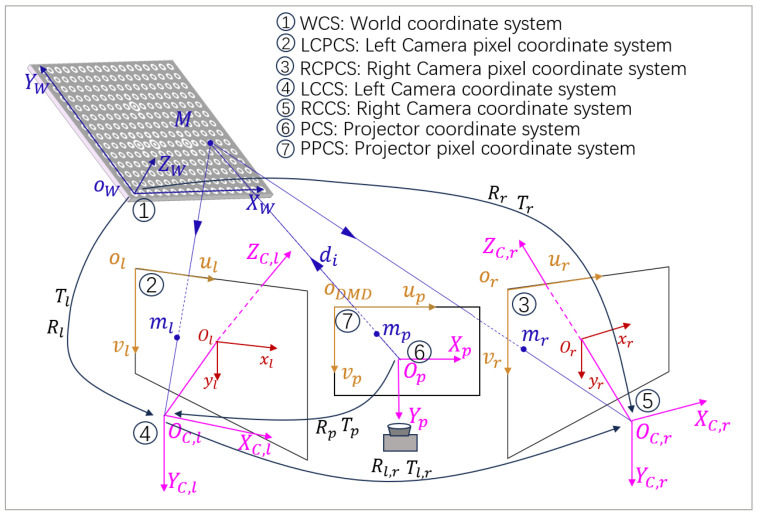
Binocular gaze cursor calibration model.

As shown in Figure 1, the binocular light model $M={\left[X_{W},Y_{W},Z_{W}\right]}^{T}$ is the coordinate of the calibration object in WCS, and then $\tilde{M}={\left[X,Y,Z,1\right]}^{T}$ is the homogeneous coordinate of M. Taking the left camera as an example, the imaging process is described as follows: $m_{l}={\left[u_{l},v_{l}\right]}^{T}$ is the ideal imaging coordinate of M in LCPCS; ${\tilde{m}}_{l}={\left[u_{l},v_{l}\right]}^{T}$ is the homogeneous coordinate of $m_{l}$; $R_{l}$ and $T_{l}$ are the 3×3 rotation matrix and 3×1 translation matrix from WCS to LCPCS, respectively, because the calibration board is a two-dimensional plane; and the $Z_{W}$ component in WCS is 0. Therefore, the relationship between $\tilde{M}$ and ${\tilde{m}}_{l}$ is

(1) $$s{\tilde{m}}_{l}=A_{l}\left[\begin{array}{ll} R_{l} & T_{l} \end{array}\right]\tilde{M}=A_{l}\left[\begin{array}{llll} r_{1,l} & r_{2,l} & r_{3,l} & T_{l} \end{array}\right]\left[\begin{array}{c} X_{l}\\ Y_{l}\\ 0\\ 1 \end{array}\right]=A_{l}\left[\begin{array}{lll} r_{1,l} & r_{2,l} & T_{l} \end{array}\right]\left[\begin{array}{c} X_{l}\\ Y_{l}\\ 1 \end{array}\right]=H_{l}\left[\begin{array}{c} X_{l}\\ Y_{l}\\ 1 \end{array}\right]$$

s is a scale factor, $A_{l}=\left[\begin{array}{ccc} f_{x,l} & 0 & u_{0,l}\\ 0 & f_{y,l} & v_{0,l}\\ 0 & 0 & 1 \end{array}\right]$ is the 3×3 intrinsic matrix of the left camera, where $\left(u_{0,l},v_{0,l}\right)$ is the principal point of the camera, and $f_{x,l}$ and $f_{y,l}$ are the scale factors of the left camera in the u and v directions. $H_{l}$ is the homography matrix, which can be obtained through four calibration objects in the same plane.

Given three or more homography matrices, the intrinsic parameters of the camera and the rotation translation matrix corresponding to each rotation matrix can be determined. Please refer to reference [19] for detailed calculation methods.

Camera lenses have distortion, and the distortion model is

(2) $$\left\{\begin{array}{l} x_{d,l}=x+x\left(k_{1,l}r^{2}+k_{2,l}r^{4}+k_{3,l}r^{6}\right)+2p_{1,l}y+p_{2,l}\left(r^{2}+2x^{2}\right)\\ y_{d,l}=y+y\left(k_{1,l}r^{2}+k_{2,l}r^{4}+k_{3,l}r^{6}\right)+2p_{1,l}x+p_{2,l}\left(r^{2}+2y^{2}\right) \end{array}\right.$$

$\left(x,y\right)$ and $\left(x_{d,l},y_{d,l}\right)$ are the image coordinates before and after distortion; $k_{1,l}$, $k_{2,l}$, and $k_{3,l}$ are radial distortion coefficients; and $p_{1,l}$ and $p_{2,l}$ are the tangential distortion coefficients of the camera. Similarly, the right camera’s intrinsic matrix, rotation matrix, translation matrix, and distortion coefficients can be calculated.

As shown in Figure 1, LCCS can be transformed to RCCS using *R* and *T*.

(3) $$\left\{\begin{array}{l} R_{l,r}=R_{r}^{i}{\left(R_{l}^{i}\right)}^{-1}\\ T_{l,r}=T_{r}^{i}-R_{r}^{i}{\left(R_{l}^{i}\right)}^{-1}T_{l}^{i} \end{array}\right.$$

The projector does not calibrate conventional binocular structured light 3D measurement as an independent unit. However, to achieve pixel-level fill light in this paper, the rotation and translation matrices $R_{p}$, $T_{p}$ relative to the camera and the projected coordinates $m_{p}$ on the DMD need to be obtained. See reference [20] for specific procedures.

## 3. Pixel-Level Adaptive Fill Light Principle

Using emitting pixels A and B on the DMD, as shown in Figure 1, the principle of fill light is demonstrated. $A^{\prime }$ and $B^{\prime }$ represent the projections of the emitting pixels on the calibration board plane. $d_{A}$ and $d_{B}$ indicate the light propagation distances of the emitting pixels. In the three-dimensional world, it can be represented by $d_{i}$ in Figure 1. The different $d_{A}$ and $d_{B}$ cause the light emitted by A and B with the same brightness to have varying light intensities when reaching the calibration board plane after attenuation, brighter at the near end and dimmer at the far end. This affects the accuracy of m in Section 2 and the calibration results. This section fully utilizes binocular structured light projectors’ programmable light intensity feature to achieve pixel-level fill light for the calibration board. The essence is to calculate the distances $d_{A}$ and $d_{B}$ and adjust the pixel brightness of points A and B on the DMD based on the distance–light intensity relationship, ensuring that the light received by $A^{\prime }$ and $B^{\prime }$ on the calibration board plane is uniform. Firstly, determine the intersection line $L_{1}$ between the DMD and the calibration board plane and the angle between the two planes θ. Then, use the inverse camera method to find the pixel positions A and B of the tilted calibration pattern on the projector DMD, and calculate the vertical distance from $A^{\prime }$ to the DMD plane, denoted as $h_{A}$. Utilize geometric relationships in $\Delta A^{\prime }AL_{1}$ to calculate $d_{A}$ and similarly for $d_{B}$. Finally, the pixel brightness along the tilted direction is determined based on the relationship between light intensity attenuation and distance. Reproject the calculated image onto the calibration board to achieve uniform illumination. The detailed process is as follows.

### 3.1. Inverse Camera Projection

For pixel-level light intensity control, knowing the position of the calibration object on the projector’s DMD chip is essential. The projector cannot directly capture the calibration board pattern as an output device. However, the internal camera parameters and the rotation–translation matrix relative to the camera can be obtained using the inverse camera method [20], allowing the calibration object’s position on the projector’s DMD to be determined through coordinate transformations. The process is as follows:

(4) $$s\left[\begin{array}{c} u_{p}\\ v_{p}\\ 1 \end{array}\right]=A_{p}{\left[\begin{array}{cc} R_{p} & T_{p}\\ 0 & 1 \end{array}\right]}^{-1}\left[\begin{array}{cc} R_{l}^{i} & T_{l}^{i}\\ 0 & 1 \end{array}\right]\left[\begin{array}{c} X_{w}\\ Y_{w}\\ Z_{w}\\ 1 \end{array}\right]$$

where $\left(u_{p},v_{p}\right)$ represents the coordinates of the calibration object on the DMD and s is the scale factor. The intrinsic matrix of the projector, denoted as $A_{p}$, includes the focal length, pixel size, and the coordinates of the principal point; $R_{p}$ and $T_{p}$ are the rotation and translation matrices of the projector relative to the camera; and $R_{l}^{i}$ and $T_{l}^{i}$ are the rotation and translation matrices of the calibration board relative to the left camera, both derived from the perspective matrix H and the internal parameter matrix A [6].

### 3.2. Calculate Attenuation Distance

**Figure 2:**
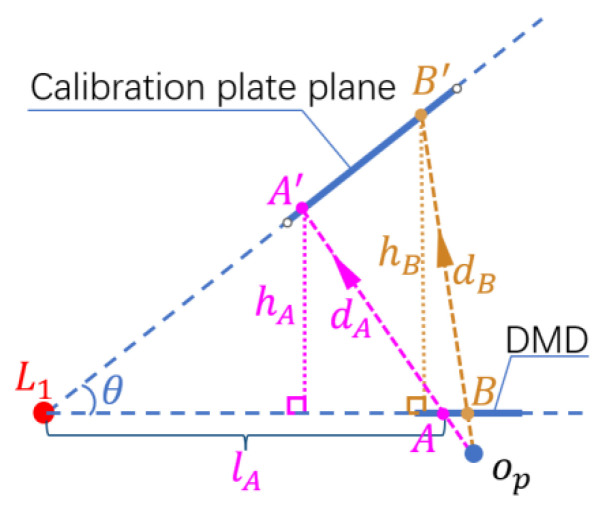
Adaptive fill light principle diagram.

Find the tilt direction of the calibration object on the DMD, which is perpendicular to the intersection line of the calibration plane and the projection plane, as each position on the calibration board shown in Figure 2 gradually changes its distance to the DMD along the slant direction. The process of solving the intersection line is as follows:

First, solve for representing a set of orthogonal unit vectors on the rotated calibration board.

(5) $$\left\{\begin{array}{c} A=[-\sin\tau \cdot \cos\beta \;l_{A}\cdot \cos\left(\arctan\frac{\sin\tau \cdot \sin\beta }{\cos\tau }+\alpha \right)\;l_{A}\cdot \sin\left(\arctan\frac{\sin\tau \cdot \sin\beta }{\cos\tau }+\alpha \right)]\\ B=[\cos\tau \cdot \cos\beta \;l_{B}\cdot \cos\left(\arctan\frac{\cos\tau \cdot \sin\beta }{\sin\tau }-\alpha \right)\;-l_{B}\cdot \sin\left(\arctan\frac{\cos\tau \cdot \sin\beta }{\sin\tau }-\alpha \right)] \end{array}\right.$$

where $l_{A}$ and $l_{B}$ are represented as

(6) $$\left\{\begin{array}{c} l_{A}=\sqrt{{\left(\cos\tau \right)}^{2}+{\left(\sin\tau \cdot \sin\beta \right)}^{2}}\\ l_{B}=\sqrt{{\left(\sin\tau \right)}^{2}+{\left(\cos\tau \cdot \sin\beta \right)}^{2}} \end{array}\right.$$

In Equation (5), α, β, τ represent the rotation angles of the calibration board around the projector’s ZYX axes [21,22,23]. Therefore, the expression for the calibration board plane is

(7) $$z=\frac{-1}{n_{3}}\left(n_{1}x+n_{2}y\right)+\frac{1}{n_{3}}\left(n_{1}T_{1}+n_{2}T_{2}+n_{3}T_{3}\right)$$

Let n=A×B, $T=T_{c}-T_{p}$. Set the projector DMD plane as the XY plane. By setting z=0 in Equation (3), the intersection line $L_{1}$ between the projector plane and the camera plane can be obtained.

**Figure 3:**
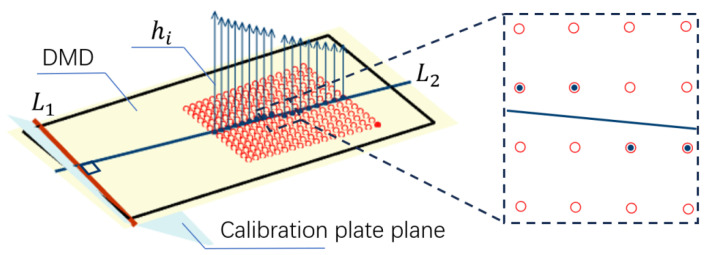
Fitting the height of the calibration board using the projections of calibration objects near $L_{2}$.

As shown in Figure 3, $L_{1}$ represents the intersection line between the calibration board plane and the DMD plane. The red dots represent the projections of the calibration objects onto the DMD plane. A line $L_{2}$ is drawn on the DMD plane, perpendicular to $L_{1}$ and passing through the projection of the calibration object. This allows us to determine that the calibration board plane is tilted along the direction of line $L_{2}$. We only need to find the distances from the calibration object projections near $L_{2}$ to the DMD, represented by $h_{i}$ in the figure, in order to fit the distance variation pattern of the entire calibration board plane relative to the DMD plane. The blue arrows in Figure 3 indicate the distances from the calibration objects near $L_{2}$ to the DMD.

Identify calibration objects near $L_{2}$, such as the black dots in Figure 3, and use trigonometry to calculate the perpendicular distance from the corresponding calibration object on the calibration board to the DMD, represented by $h_{i}$ in Figure 3. The calculation formula is as follows:

(8) $$h_{i}=\frac{f_{p}f_{l}B}{x_{p,i}f_{c}-x_{c,i}f_{p}}$$

$f_{p}$ is the projector focal length, $f_{l}$ is the left camera focal length, $x_{p,i}$ and $x_{l,i}$ are the horizontal coordinates of the calibration objects near $L_{2}$ on the DMD and camera sensor planes, and B is the baseline distance between the projector and the camera. The curve fitting function is obtained by fitting the distances from the projections of these calibration objects on the DMD to $L_{1}$ and $h_{i}$.

(9) $$h_{i}=f_{dist}(l_{i})$$

$l_{i}$ represents the distance projected to $L_{1}$ for the purpose of calculating the vertical distance from any pixel on the DMD to the calibration board plane. This is necessary because pixels without calibration object projections cannot be measured using triangulation due to the lack of stereo matching.

The vertical distance $h_{i}$ shown in Figure 2 is not the optical path length. The optical path length $d_{i}$ can be calculated using geometric relationships in the triangle formed by the intersection of the optical path with the DMD plane and the camera plane, with the formula:

(10) $$d_{i}=\sqrt{{\left(\frac{h_{i}}{\tan\theta }-l_{i}\right)}^{2}+h_{i}}$$

θ is the angle between the calibration board plane and the DMD.

### 3.3. Draw the Fill Light Pattern

Adjust the brightness of each DMD pixel based on light intensity and distance. The rules for brightness adjustment are as follows:

(11) $$\frac{k_{i}}{255}\phi \frac{1}{d_{i}^{2}}=\frac{k_{\max}}{255}\phi \frac{1}{d_{\max}^{2}}$$

**Figure 4:**
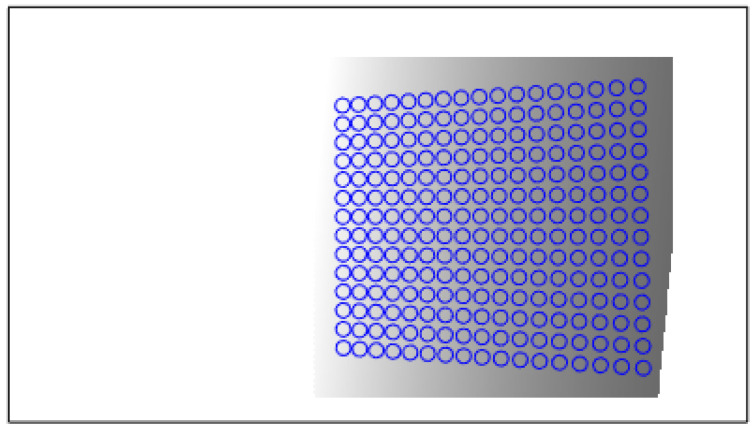
Adaptive fill light pattern projected by the projector.

$k_{i}$: grayscale value of any DMD pixel. $h_{i}$: distance from the pixel to the calibration board plane. $k_{\max}$: grayscale value of the farthest DMD pixel, usually set to 255. $h_{\max}$: distance from far end pixel to calibration board. ϕ: light intensity determined by the projector. Equation (11) adjusts the intensity to match the far end after attenuation. Find the calibration board projection area on the projector DMD and calculate the grayscale image in this area on the DMD using Equations (9)–(11), as shown in Figure 4:

The black frame represents the entire DMD target surface; the blue circle shows a projection of the calibration object on the surface (calculated, not actual), and the dashed frame displays the computed fill light pattern. Projecting the entire image back onto the calibration board ensures uniform illumination. Adaptive exposure helps achieve optimal imaging of calibration objects at both near and far ends.

## 4. The Proposed RCCPSO

Direct Linear Transformation can obtain the camera’s intrinsic and extrinsic parameters in Section 2. However, the accuracy is low, and the distortion coefficient $k_{1},k_{2},k_{3},p_{1},p_{2}$ cannot be obtained, so a nonlinear optimization method is needed. The Particle Swarm Optimization algorithm, with simple operation, high optimization accuracy, and fast operation speed, iteratively updates the position and velocity of particles to find the optimal solution. The formulas for updating particle position and velocity are as follows:

(12) $$\left\{\begin{array}{c} v_{id}^{t+1}=wv_{id}^{t}+c_{1}r_{1}\left[\;\mathrm{pbest}\;-x_{id}^{t}\right]+c_{2}r_{2}\left[\;\mathrm{gbest}\;-x_{id}^{t}\right]\\ x_{id}^{t+1}=x_{id}^{t+1}+v_{id}^{t+1} \end{array}\right.$$

$v_{id}^{t+1}$: next particle velocity, pbest: particle historical best position, gbest: best position among all particles, $x_{id}^{t}$: current particle position, $x_{id}^{t+1}$: next particle position, $c_{1}$: self-learning factor, $c_{2}$: global learning factor, and $r_{1}$ and $r_{2}$: random factors.

Taking the optimization of the left camera internal parameters and lens distortion coefficients as an example, we first establish the objective function. Assuming calibration patterns are captured from n angles, with each pattern extracting the centers of m calibration objects, the objective function can be represented as

(13) $$f_{obj}=\min\sum_{i=1}^{n}\sum_{j=1}^{m}\left\|{\hat{p}}_{ij}-p_{ij}\left(A_{l},k_{1,l},k_{2,l},k_{3,l},p_{1,l},p_{2,l},R_{l}^{i},T_{l}^{i},M_{ij}\right)\right\|$$

where $f_{obj}$ is the iteration objective and ${\hat{p}}_{ij}$ represents the pixel coordinates of the j-th calibration object in the i-th image. $p_{ij}\left(A_{l},k_{1,l},k_{2,l},k_{3,l},p_{1,l},p_{2,l},R_{l}^{i},T_{l}^{i},M_{ij}\right)$ is the projection from the calculated three-dimensional world coordinate $M_{ij}$ to the two-dimensional pixel coordinate, where $k_{1,l},k_{2,l},k_{3,l},p_{1,l},p_{2,l}$ is the lens distortion coefficient and $A_{l}$ denotes the camera intrinsic parameters. The particle’s coordinates are composed of $A_{l},k_{1,l},k_{2,l},k_{3,l},p_{1,l},p_{2,l}$. We will design a new flight method to enable the particle to accurately reach the optimal solution.

Unlike conventional Particle Swarm Optimization algorithms, this paper divides the particles into aggressive and conservative particles and presents a new flight rule. In the rest of the paper, Section 4.1 will describe how to select aggressive and conservative particles, Section 4.2 will introduce the flight rules for different particles, and finally, Section 4.3 will explain how to control the number of each type of particle during flight.

### 4.1. Particle Classification

In RCCPSO, particles are divided into aggressive and conservative types. Aggressive particles are those with better performance and meeting aggressive criteria, while the rest are considered conservative particles. Lower fitness values indicate better particle performance based on the objective function definition. Particles are sorted based on their fitness values before each iteration, as follows:

(14) $$\chi =\left\{X^{\left(1\right)},X^{\left(2\right)},\ldots ,X^{\left(K\right)},\ldots ,X^{\left(N\right)}|f_{fitness}\left(X^{\left(1\right)}\right)⩽f_{fitness}\left(X^{\left(2\right)}\right)\ldots ⩽f_{fitness}\left(X^{\left(K\right)}\right)\ldots ⩽f_{fitness}\left(X^{\left(N\right)}\right)\right\}$$

**Figure 5:**
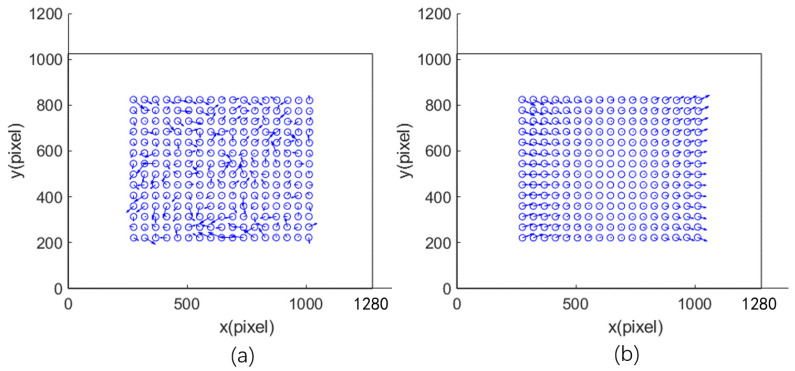
The representation of reprojection errors for different particles, where (**a**) signifies the reprojection error of conservative particles, and (**b**) indicates the reprojection error of radical particles.

χ archives store sorting information of all current particles X, and $f_{fitness}\left(X\right)$ is the fitness value of particle X, which is the average reprojection error calculated under the current particle. The top K particles are temporarily referred to as excellent particles, where K is the threshold for aggressive particles. Excellent particles have lower reprojection errors, but the error patterns differ significantly among particles. For example, as shown in Figure 5, the reprojection errors with the influence of two excellent particles have the same average value in Figure 5a,b. However, the reprojection errors in Figure 5b exhibit a more regular pattern, caused by the overshoot of distortion coefficient $k_{1,l}$. Consequently, there must be an un-overshoot $k_{1,l}$ in the continuous domain around the particles in Figure 5b, resulting in lower average reprojection errors than in Figure 5a. Similarly, overshooting of other optimized parameters will lead to the regularization of reprojection error directions. Thus, we define the error direction chaos coefficient using Equation (15):

(15) $$ChaosCoeff=\sum_{i=1}^{s}\sum_{j=2}^{t-1}\frac{\left(A_{i,j}-A_{i,j-1}\right)\left(A_{i,j+1}-A_{i,j}\right)}{\left|\left(A_{i,j}-A_{i,j-1}\right)\left(A_{i,j+1}-A_{i,j}\right)\right|}$$

Here, $A_{i,j}$ denotes the reprojection error direction angle for the calibration object at the *i*-th row and *j*-th column, with s and t being the total rows and columns of the calibration object. ChaosCoeff is the chaos coefficient. If ChaosCoeff<0, it means the unordered variation of $A_{i,j}$ is greater than the patterned variation, indicating the reprojection error direction is generally random and the particle is aggressive; otherwise, it is conservative.

### 4.2. Flight Rules Design for Aggressive and Conservative Particles

Design different guidance factors and learning weights for different particles to guide their flight, with velocity update rules as shown in the equation.

(16) $$v_{id}^{t+1}=wv_{id}^{t}+c_{1}r_{1}\left[\;E_{i}-x_{id}^{t}\right]+c_{2}r_{2}\left[\;\mathrm{Gbest}\;-x_{id}^{t}\right]$$

In a typical particle swarm, all particles flying toward Gbest may lead to loss of population diversity, premature convergence, and stagnation. When a particle is identified as aggressive, it performs well and has better solutions nearby. Elite particles should take on more exploration tasks, rather than converging to the global optimum. To do this, elite particles need to reduce the attraction to Gbest, become more confident, and increase self-awareness. Therefore, the international learning factor $c_{2}=0$ and the self-learning factor is

(17) $$E_{i}=Pbest_{i}$$

**Figure 6:**
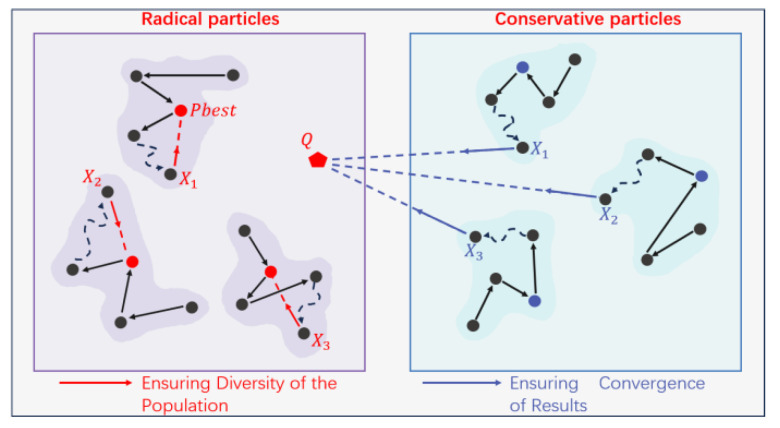
Illustration of flight modes for different particles.

As shown in Figure 6, the red arrows indicate elite particles flying in multiple directions, promoting population exploration and enhancing diversity.

Using conservative particles to explore space is unrealistic as their solutions are lower quality than aggressive particles, and better solutions around them are unknown. However, they can be used to move towards promising regions around aggressive particles to enhance the local search capability of aggressive particles. Therefore, this paper designs the guidance factor for conservative particles as the weighted center of the aggressive particles $Pbest_{i}$. The weight values are determined based on the fitness values of each aggressive particle. This way, the weighted center contains high-quality information, providing a hopeful direction for conservative particles. The specific operation is as follows:

(a) First, calculate the weight for each aggressive particle, with the weight value determined by its fitness value according to Equation (18). The fitness value $f_{fitness}\left(X_{i}\right)$ of the i aggressive particle is used, where a smaller fitness value results in a more considerable weight.

(18) $$W(i)=\frac{1}{1+f_{fitness}\left(X_{i}\right)}$$

(b) Create the weighted guidance center for aggressive particles, where Q is the guidance center generated by aggressive particles, ${\mathrm{Pbest}\;}_{k}$ is the k-th aggressive particle, and M is the total number of aggressive particles in the current iteration.

(19) $$Q=\sum_{k=1}^{M}\frac{W(k)\cdot {\;\mathrm{Pbest}\;}_{k}}{\sum_{k=1}^{N}W(k)}$$

(c) Let the weighted guidance center of aggressive particles guide conservative particles. The weighted center, as shown in Figure 6, contains beneficial information among aggressive particles. This region is necessary for exploration by conservative particles.

(20) $$E_{i}=Q$$

### 4.3. Controlling the Number of Aggressive Particles

In the early stages of iteration, to maintain population diversity and enhance exploration capability, the threshold for generating aggressive particles must be lowered, and the number of conservative particles must be decreased. As iteration progresses and valuable regions become more apparent, the transition from exploration to exploitation occurs by raising the threshold for aggressive particles and increasing the number of conservative particles. This paper proposes a dynamic management approach for aggressive particles by gradually raising the thresholds for aggressive and conservative particles as iterations increase to reduce the number of aggressive particles. Calculate threshold K based on Equation (21).

(21) $$K=\left\lceil N*R_{\max}-\left(N*R_{\max}-N*R_{\min\;}\right)\cdot \left(\frac{\;\mathrm{iter}\;}{\;\mathrm{Maxiteration}\;}\right)\right\rceil \;$$

where $\left\lceil a\right\rceil$ is the floor function, $R_{\max}$ is the maximum threshold ratio of the total population, $R_{\min}$ is the minimum ratio, N is the total number of particles, iter is the current iteration number, and Maxiteration is the maximum number of iterations. As the iteration number increases, the threshold for aggressive particles linearly rises, and their quantity gradually decreases. This is beneficial for maintaining diversity in the early stages and exploitation in the later stages.

The pseudocode implementation of RCCPSO is as follows (See Algorithm 1):

**Algorithm 1:** RCCPSO

$\;Input:\;A_{l},k_{1,l},k_{2,l},k_{3,l},p_{1,l},p_{2,l},R_{l}^{i},T_{l}^{i},M_{ij}$



$\;Output:\;\mathrm{Optimized}\;\mathrm{parameters}:A_{l},k_{1,l},k_{2,l},k_{3,l},p_{1,l},p_{2,l}$



    begin



$\;particle=\left[A_{l},k_{1,l},k_{2,l},k_{3,l},p_{1,l},p_{2,l}\right];$



        // Randomly initialize N particles 



$\;particle\left[1,\ldots ,N\right]=\mathrm{Randomize}\;\mathrm{Particles}\left(particle\right);\;\;$



        while iter<ger do 



$\;//\;\mathrm{Step}.1\;\mathrm{Get}\;\mathrm{Threshold}\;K\;\mathrm{according}\;\mathrm{to}\;\mathrm{Equation}\left(21\right)\;$



$\;K=\mathrm{GetParticlesThreshold}\left(iter,ger,N\right);$



            // Step.2 Calculate the fitness of the N particles 



$\;finess\left[1,\ldots ,N\right]=\mathrm{GetParticlesFitness}\left(particle\right);\;$



$\;//\mathrm{Step}.3{\;\mathrm{Sort}\;\mathrm{particle}}_{i}\mathrm{for}\;l<i<N\;\mathrm{according}\;\mathrm{to}\;\mathrm{Equation}\left(14\right)\;\;$



$\;particle\left[1,\ldots K,\ldots ,N\right]=\mathrm{Sort}\;\mathrm{Particles}\left(finess,K\right);\;$



              // Step.4 Classify the particles into Radical particles and Conservative 



$\;\mathrm{particles}\;\mathrm{according}\;\mathrm{to}\;\mathrm{Equation}\left(15\right)\;$



$\;RadicalParticles=\mathrm{PickRadicalParticles}\left(particle\left[1,\ldots ,K\right]\right);\;$



              ConservativeParticles=Particles−RadicalParticles;  



$\;//\;\mathrm{Step}.5\;\mathrm{Update}\;\mathrm{the}\;\mathrm{flight}\;\mathrm{rules}\;\mathrm{of}\;\mathrm{particles}\;\mathrm{according}\;\mathrm{to}\;\mathrm{Equations}\left(17\right)–\left(20\right)\;$



$\;\left[E_{c},E_{r}\right]=\mathrm{UpdateFlightRules}\left(RadicalParticles\right);\;$



$\;//\;\mathrm{Step}.6\;\mathrm{Update}\;\mathrm{the}\;\mathrm{flight}\;\mathrm{rules}\;\mathrm{of}\;\mathrm{particlesaccording}\;\mathrm{to}\;\mathrm{Equations}\left(12\right)\mathrm{and}\left(16\right)\;$



$\;Particles=\mathrm{UpdateParticlePosition}\left(E_{c},E_{r},ConservativeParticles,RadicalParticles\right);\;$



        end  



        // Find the particle with the smallest fitness 



$\;BestParticles=\mathrm{Min}\left(Particles,\;finess\right);\;$



$\;\mathrm{return}\;\left[A_{l},k_{1,l},k_{2,l},k_{3,l},p_{1,l},p_{2,l}\right]=BestParticles;\;$



    end



**Figure 7:**
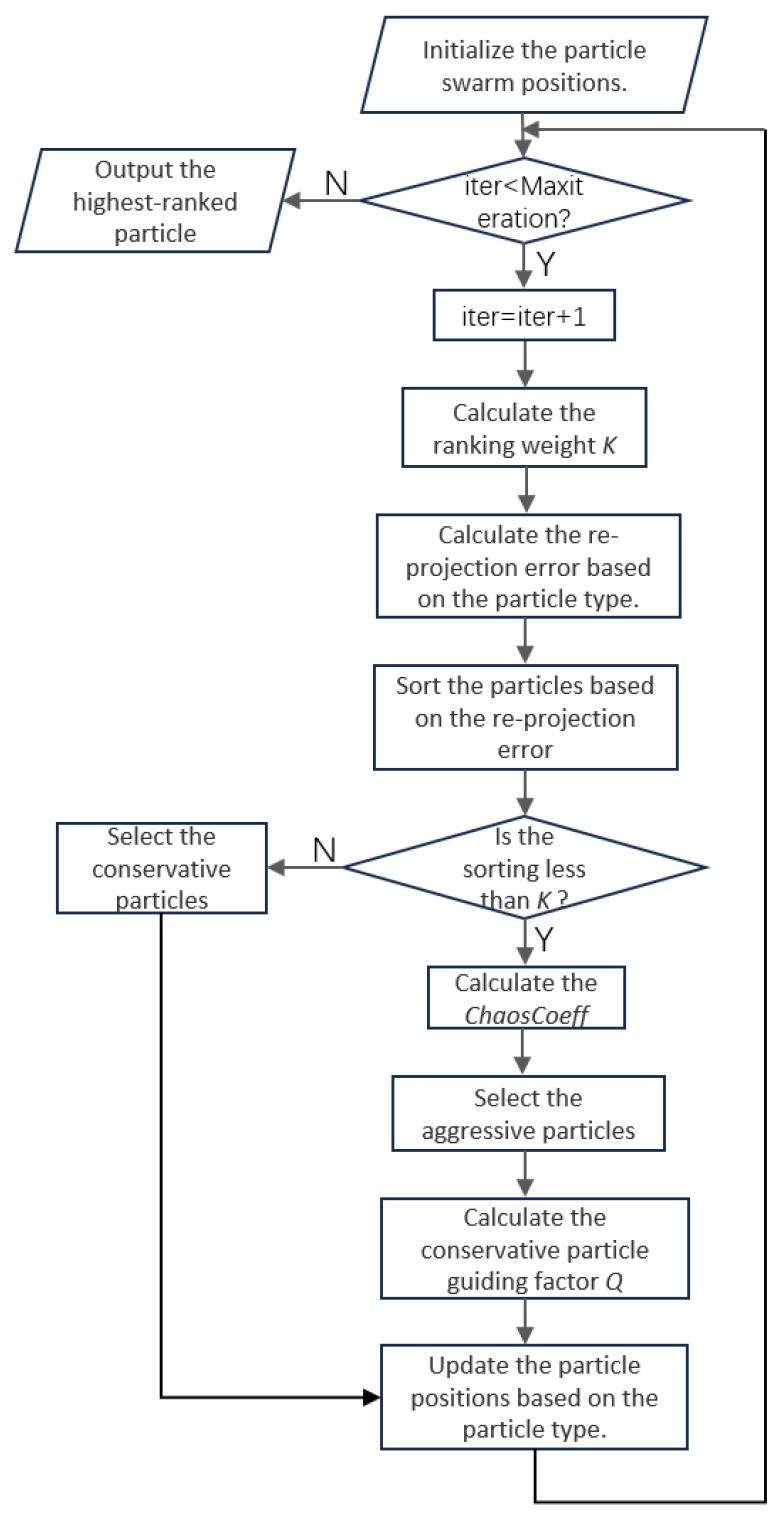
The algorithm flow of RCCPSO.

The algorithm flow of RCCPSO is shown in Figure 7.

## 5. Experimental Results and Analysis

**Figure 8:**
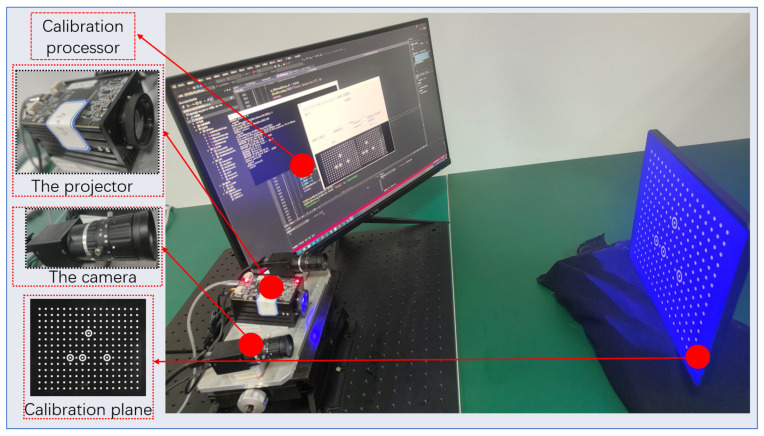
Stereo vision calibration experiment platform.

We conducted a comparative experiment to validate the improved stereo vision calibration method for accuracy. We used two MV-CA013-20GM(Hangzhou Hikvision Robotics Co., Ltd., Hangzhou, China) cameras and DLP3010(Shanghai Zhengyuan Technology Co., Ltd., Shanghai, China) for the structured light platform. The camera lens focal length was 12 mm. The image resolution was 1280 × 1080. The pixel size was 4.8 μm×4.8 μm. The experiment was carried out on Windows 10 64-bit, 2.30 GHz, 8 GB RAM, MATLAB 2020b. We utilized a cc-250-300-15*19 high-precision marble calibration board (Cangzhou Borun Measuring Instrument Co., Ltd., Cangzhou, China) to reduce the manufacturing impact. The experimental environment is shown in Figure 8.

**Figure 9:**
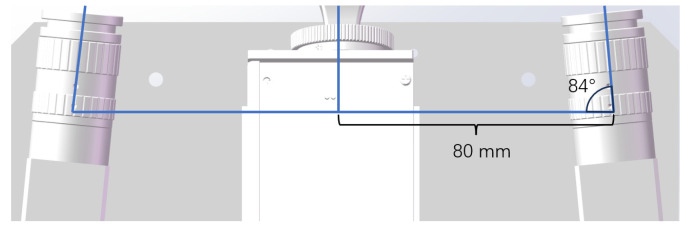
Schematic diagram of the experimental setup.

We present the mechanical parameters of the experimental setup in Figure 9, where the optical centers of the two cameras and the projector are kept as close as possible to the same plane. The angle between the main optical axis of the camera and that of the projector is approximately 86°, and the distance between the optical centers of the camera and the projector is about 80 mm.

### 5.1. Adaptive Fill Light System Algorithm Verification

Four sets of comparative calibration experiments were conducted in this section to validate the adaptive fill light algorithm’s effectiveness in improving calibration accuracy. The first set used directly captured images for calibration; the second used images captured after adaptive exposure. The images needed for the third set were obtained by gamma, which corrected the photos captured in the first set. The images required for the fourth set were captured after adaptive fill light. Images for the first, second, and fourth sets were captured simultaneously. The images needed for the first set were captured first, maintaining the calibration board’s position before capturing pictures required for the second and fourth sets. Each set underwent 25 calibration experiments, with each experiment capturing 15 pairs of calibration patterns with the left and right cameras. The calibration object coordinates were extracted using the same method and imported into MATLAB2016 to calculate the calibration results. The first group and the fourth group of experiments both captured images with an exposure time of 15 ms.

**Figure 10:**
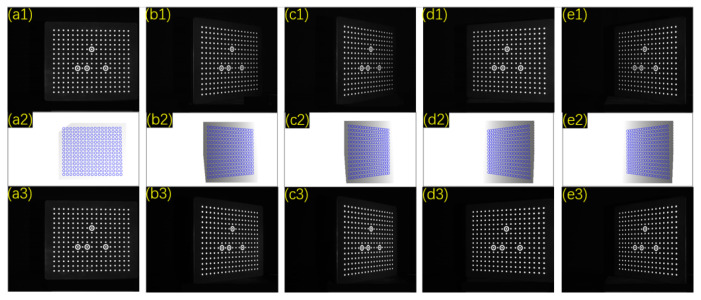
Partial patterns collected in the calibration experiment. (**a1**–**e3**) represent five different poses of the calibration board.

**Figure 11:**
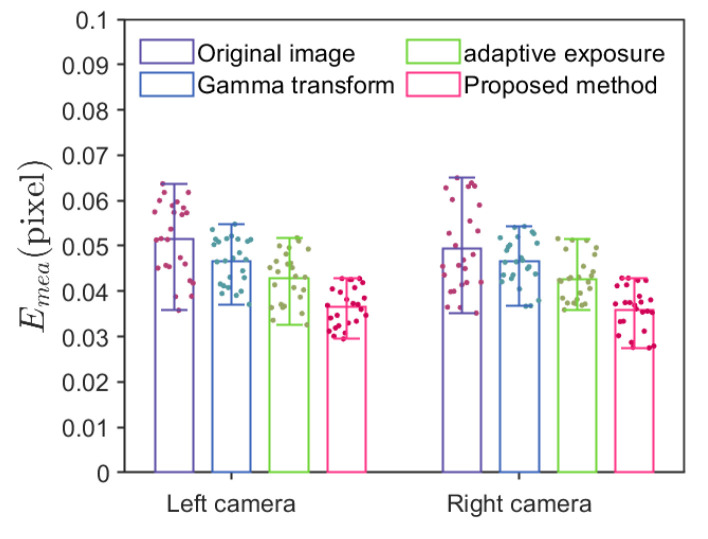
Reprojection error plot.

Figure 10 shows selected calibration patterns. Image 1 is the calibration board image before the fill light, image 2 is the fill light image after the adaptive fill light algorithm, and image 3 is the image after the fill light. Improvements in shadows b, c, d, and e are noticeable after adaptive fill light. The calibration experiments on four sets of images yielded the reprojection error plot shown in Figure 11. The height of the bars in the plot represents the average reprojection error for each experiment, and the colored dots represent the reprojection error values for each experiment. Unprocessed images have the highest reprojection error, with left and right camera errors of 0.0515 and 0.0495 pixels. Images captured after adaptive fill light have the lowest reprojection error of 0.0365 and 0.0362 pixels, representing a 28% average decrease compared to the original images. Calibration using images processed with adaptive gamma correction and exposure results in lower reprojection errors than the original images but higher than the proposed method in this paper. Regarding data stability, this paper’s method outperforms the other experimental groups. Using standard deviation to measure data stability, the first group’s left and right reprojection error standard deviations are 0.00828 and 0.00971 pixels. The fourth group’s deviations are 0.00437 and 0.00495 pixels, representing an average decrease of 48.154% compared to the first group. The second and third groups have an average standard error for the left and right cameras of 0.00523 and 0.00502 pixels, lower than the first group but higher than the fourth group, with a slight difference. Overall, the proposed method achieves higher and more stable calibration accuracy.

**Figure 12:**
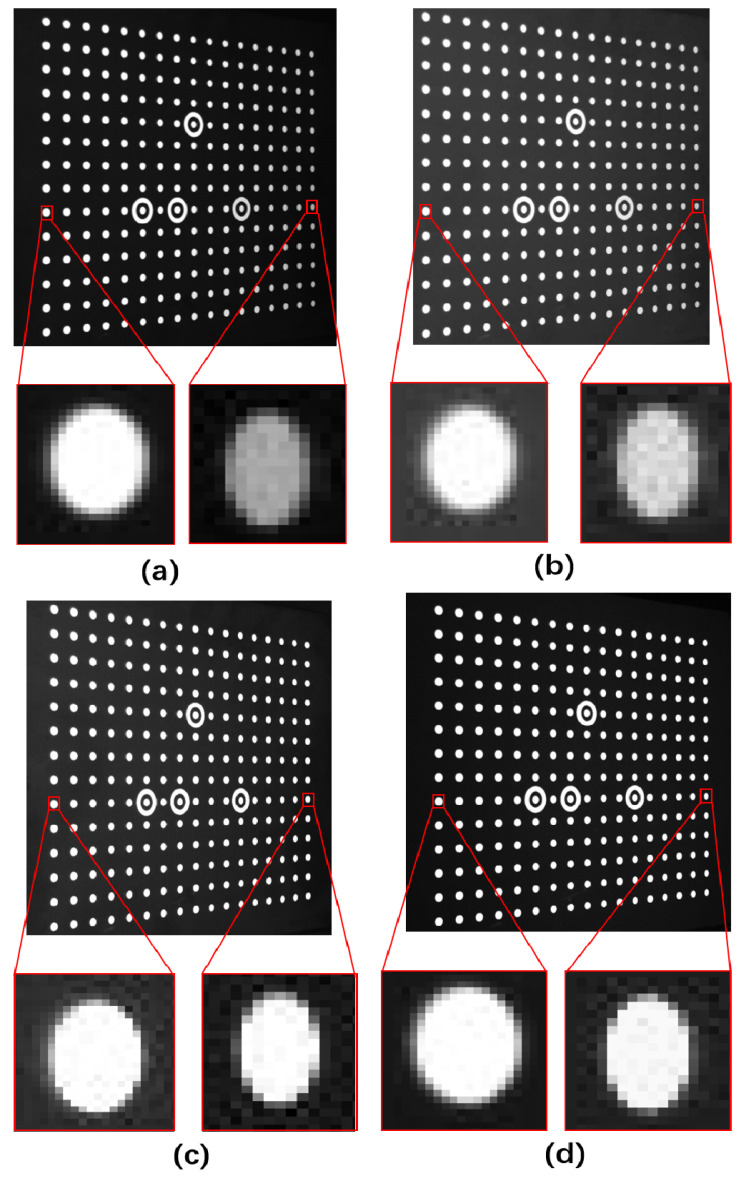
Zoom-in Image. (**a**–**d**) represent the sharpness of the calibration object’s edges under four methods: original image, gamma adjustment, adaptive exposure, and pixel-level dimming.

**Figure 13:**
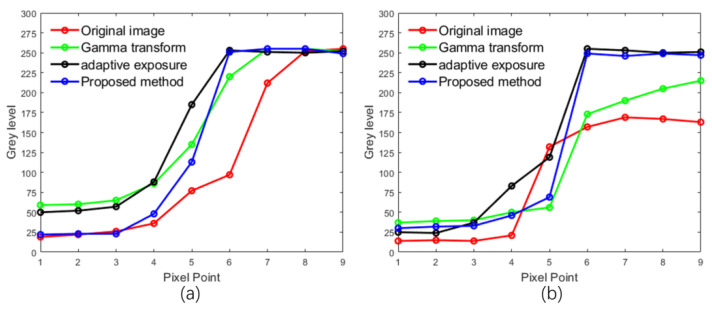
Edge pixel variation diagram. (**a**) Proximal pixel variation, (**b**) distal pixel variation.

**Figure 14:**
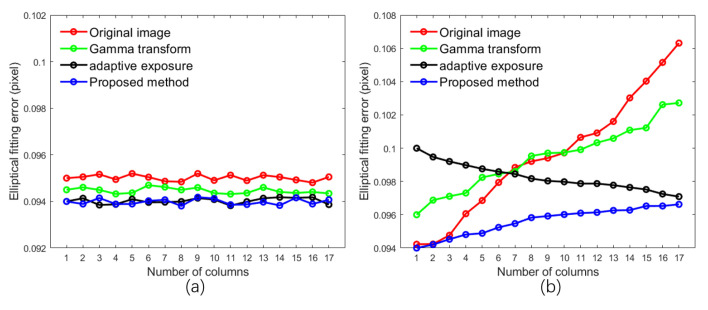
Relationship between column number of calibration object and ellipse fitting error. (**a**,**b**) represent the ellipse fitting error when the calibration board is facing the camera directly and when it is tilted towards the camera, respectively.

To analyze how our method impacts captured images and calibration results, we zoomed in on pictures taken at the same angle as Figure 10c in each experimental group to create Figure 12. Figure 12 shows zoomed-in areas of calibration objects near and far from the camera. In Figure 12a, distant white objects appear gray under normal lighting due to light attenuation, with blurry edges, while nearby objects are transparent. After adaptive gamma correction, Figure 12b shows increased brightness in distant objects and raises overall background brightness, causing blurry edges for nearby objects. After adaptive exposure adjustment, Figure 12c enhances dark details by increasing exposure time, resulting in precise edges for distant objects and brightening the dark background of nearby objects, making edges blurry. Figure 12d, captured with an adaptive fill light algorithm, shows precise edges for near and far objects compared to a, b, and c. Extracting edge pixels of magnified calibration objects in Figure 12a–d allows for quantitative analysis of edge clarity, producing edge grayscale images as shown in Figure 13 and Figure 14.

In Figure 13a, the pixel values transitioned to around 250. The first two groups jumped 4 pixels from below 75 to 250, while the third and fourth groups only jumped 2 pixels. The starting grayscale of the third group (black background) was around 50, higher than the fourth group. Thus, the fourth group experiment excelled for edge sharpness from the near end. Figure 13b shows the grayscale changes in the edges of distant calibration objects. All groups started at around 25, but only the third and fourth groups reached around 250. The third and fourth groups excelled for edge sharpness from the distant end. Overall, after adaptive fill light, image clarity surpassed other algorithms.

The sharpness of the calibration object’s edges directly impacts the positioning accuracy of its projected center, a crucial input for the calibration algorithm, affecting the calibration results. This analysis focuses on individual calibration objects, and next, we will extend it to the entire calibration plane. Using the ellipse fitting method to determine the projection center of the calibration object in this paper, we define the fitting error of the ellipse as

(22) $$\sigma =\sqrt{\frac{1}{n}\sum_{i=1}^{n}{\left(l_{i}-\bar{l}\right)}^{2}}$$

where n represents the number of pixels involved in ellipse fitting, $l_{i}$ denotes the distance from a pixel to the fitted ellipse, and $\bar{l}$ represents the average distance of all pixels involved in fitting to the fitted ellipse. The fitting error reflects the positioning accuracy of the calibration object, with a smaller value indicating higher precision. Precise edges of the calibration object facilitate the calculation of the actual ellipse boundary, thereby reducing the fitting error. Two images were selected from each experimental group, similar to Figure 10a,c, with the same shooting angle. The average fitting error for each column of calibration objects in the images was calculated to generate Figure 14, where the *x*-axis represents the number of columns of calibration objects, and the *y*-axis represents the average fitting error for each column. As shown in Figure 14a, when the calibration board faces the camera directly, the fitting error of the second, third, and fourth groups is lower than that of the first group, with the third and fourth groups have lower errors than the second group but have a minimal difference between the third and fourth groups. This indicates that the proposed method performs similarly well to adaptive exposure and surpasses adaptive gamma correction when handling the calibration board facing the camera directly. When the calibration board is tilted, as illustrated in Figure 14b, as the number of columns of calibration objects increases, the objects gradually move further away from the camera. The fitting error of the first and second groups increases with the number of columns. In contrast, the fitting error of the third group decreases gradually as the number of columns increases. However, the fitting error for the first seven columns is higher than that of the first, second, and fourth groups. Although the fitting error of the fourth group also increases with the number of columns, the rate of increase is slower compared to the first and second groups, with an overall lower fitting error than the third group. Through the analysis of Figure 14, it is evident that the main focus of this study is to enhance the positioning accuracy of calibration objects on tilted calibration boards to improve overall calibration accuracy.

Our approach effectively considers the lighting conditions of calibration objects at different positions on the calibration board. In contrast to global algorithms such as gamma correction and exposure time adjustment, our method enhances dark details without compromising bright information, making it an accurate adaptive fill light technique. However, in Figure 14b, our method does not exhibit a linear relationship, as the fitting error still shows an increasing trend. This may be attributed to decreased pixel details due to the smaller imaging of distant calibration objects. Although our method performs similarly to adaptive exposure in Figure 14a, it is essential to note that only a few patterns in the image group used for calibration are parallel to the camera plane, which explains why the overall calibration error in our method outperforms other approaches in the experimental results.

### 5.2. Validation of Nonlinear Optimization Algorithm

**Figure 15:**
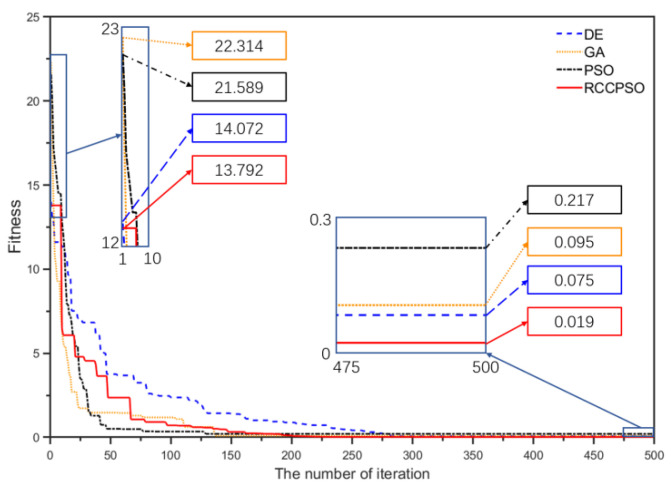
Convergence performance of four optimization algorithms.

Initially, the camera parameters were linearly computed using the Direct Linear Transformation method on the images captured in the first calibration experiment of the fourth group, without considering camera lens distortion. Subsequently, the linear solution was used as the initial solution for the RCCPSO algorithm, with the mean reprojection error as the fitness value (the smaller the calibration reprojection error mean, the better the nonlinear optimization effect) for iteration. The Particle Swarm Optimization (PSO) algorithm, Genetic Algorithm (GA), Differential Evolution Algorithm (DE), and RCCPSO algorithm were separately employed for nonlinear optimization of the camera’s linear parameters. The optimization results are shown in Figure 15. In RCCPSO, the $R_{\max}$ is set to 40% and the $R_{\min}$ is set to 10%. Both the maximum number of iterations and the population size are 500. The iteration count and population size for the other three comparison methods are consistent with those of RCCPSO.

As shown in Figure 15, after 500 iterations, the fitness value (reprojection error) of RCCPSO is 0.019, which is significantly lower than that of DE, GA, and PSO. However, in terms of convergence speed, RCCPSO is somewhat slower than PSO. PSO stabilizes after 150 iterations, while RCCPSO only becomes stable after 200 iterations. Nevertheless, the final result of RCCPSO is superior to that of PSO. This indicates that although PSO converges about 25% faster than RCCPSO, it becomes trapped in a local optimum, resulting in a 91% loss in accuracy. This is a trade-off that is not worthwhile in the high-precision structured light field.

**Figure 16:**
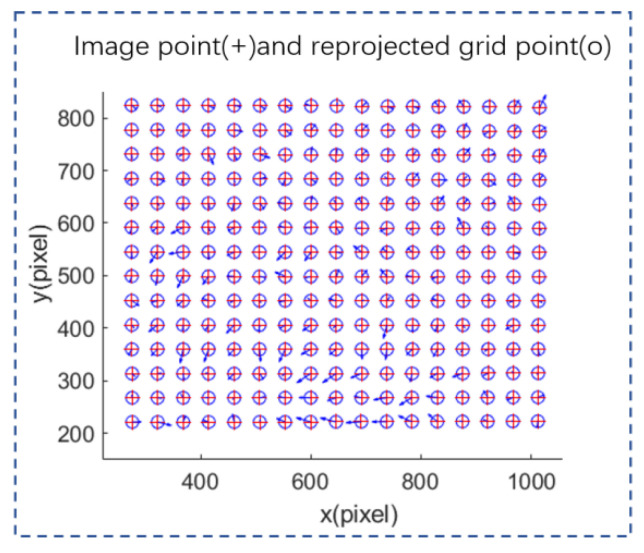
Relationship between actual points and reprojected points.

Recompute the reprojected coordinates of one of the images using the camera parameters optimized by RCCPSO and display the calculated projected coordinates along with the actual coordinates in Figure 16, where the arrow length represents the error magnitude and the arrow direction indicates the deviation between the reprojected and exact coordinates. It can be observed that the projected points calculated with the nonlinear optimization proposed in this paper closely match the actual points, demonstrating high optimization accuracy.

### 5.3. Comparison of Different Calibration Methods

This paper comprehensively improved the calibration method and compared it with Zhang’s calibration method, the Direct Linear Calibration (DLT) method, and the calibration results from the calibration toolbox. The calibration accuracy of each method is compared in Table 1, where each method was tested 25 times. “Average error” in the table represents the average reprojection error from the 25 calibration experiments, and “SE” indicates the standard deviation of the calibration errors. The calibration method improved in this paper outperformed the other three methods in terms of average error, maximum error, and the dispersion of errors.

We measured the average time consumed by four methods over 25 experiments, as shown in Table 2. Our method is less time-efficient, taking 11.8 s longer than the DLT algorithm without nonlinear optimization. However, this does not impact the method’s practicality in the field. The method achieves lower reconstruction errors (refer to 5.4), and the additional calibration time does not affect the 3D reconstruction time but improves the reconstruction accuracy with each use.

### 5.4. Structured Light 3D Reconstruction Experiment

**Figure 17:**
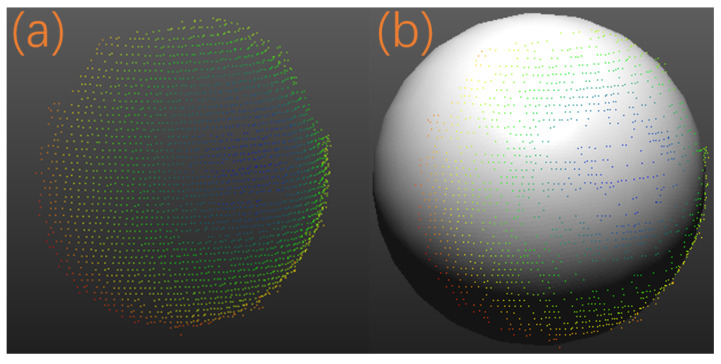
Standard sphere 3D reconstruction results. (**a**) is the 3D point cloud, and (**b**) is the point cloud reconstruction result.

To validate that the stereo-structured light system calibrated by the method in this paper can produce more precise and stable 3D reconstruction results, we used the camera’s intrinsic and extrinsic parameters obtained from the experiments in Section 5.3 as the parameters needed for this section’s experiment. Employing the stereo-structured light setup shown in Figure 8, we conducted 20 measurements on standard spheres, each varying the position and orientation of the standard sphere relative to the camera. The point clouds obtained from each measurement were fitted to spheres, and the fitted sphere radii were compared with the standard sphere radii to calculate the measurement errors (see Figure 17).

**Figure 18:**
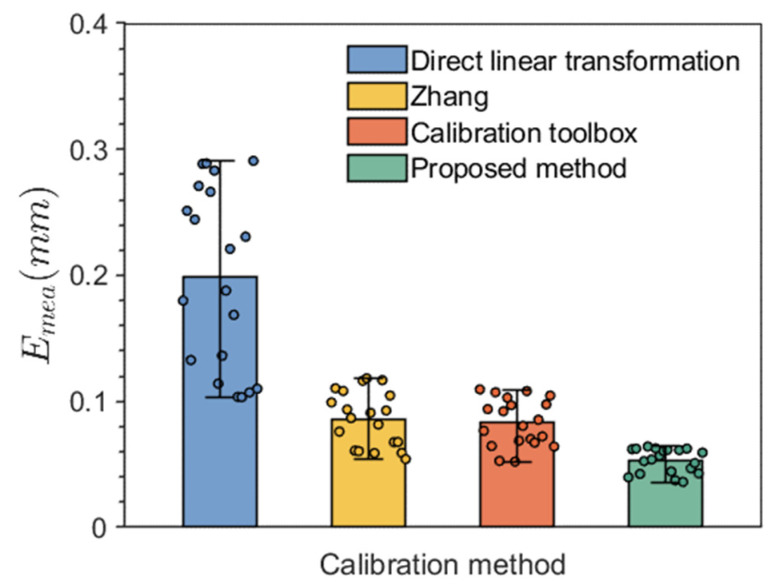
Three-dimensional reconstruction errors.

As shown in Figure 18, from the average error values, the device reconstruction error from the direct transformation method is the largest, at 0.201 mm. Zhang’s calibration method and the calibration toolbox method have the next largest errors, 0.091 mm and 0.097 mm, respectively, which are nearly identical. In contrast, the device calibration error using the method proposed in this paper has the smallest average reconstruction error, at 0.053 mm. This is because the direct transformation method cannot calibrate the lens distortion coefficients, and without calibrating these coefficients, the reconstruction error is significantly increased. Although Zhang’s method and the calibration toolbox method employ traditional nonlinear optimization algorithms to optimize parameters, they lack the optimization measures for calibration images that the proposed method includes, resulting in higher reconstruction errors. In terms of data accuracy, the reconstruction error of the proposed method is more concentrated, with higher accuracy. This is because the improved Particle Swarm Optimization algorithm proposed in this paper benefits from a synergistic flight strategy, making it easier to find the global optimal solution. In contrast, traditional methods are more susceptible to local optima, leading to error fluctuations as the standard sphere position changes.

## 6. Discussion

In calibration, binocular structured light systems typically use additional lighting to improve board illumination, which is challenging in industrial settings. This paper innovatively uses the system’s projector to optimize ambient light with programmable intensity for pixel-level adjustment. The projector also has various other calibration applications, potentially inspiring new uses. Additionally, our method incorporates reprojection error direction into nonlinear optimization, achieving a 50 µm reconstruction error at a 50 cm working distance, meeting high-precision 3D reconstruction needs in fields like industrial inspection and reverse engineering [24,25].

## 7. Conclusions

Considering the impact of calibration image quality and nonlinear optimization of intrinsic camera parameters on the calibration accuracy in the stereo-structured light calibration process, a comprehensive improvement method is proposed to enhance the calibration effect. In this work, compared with traditional calibration processes, the programmable light intensity of the projector is fully utilized to calculate the light emitted by each pixel on the DMD and the light path distance to the calibration board, and then, based on the attenuation relationship of light intensity with propagation distance, the brightness of each pixel on the DMD is calculated to evenly distribute the light intensity on the inclined calibration board, thus improving the image quality. In the nonlinear optimization stage, the RCCPSO algorithm optimizes the camera parameters. This method classifies particles into aggressive and conservative particles based on the reprojection error and designs different flight rules for other particles to utilize the advantages of aggressive particles fully. From the final 3D reconstruction accuracy perspective, our method achieves a reconstruction error of 0.0531 mm, showing a 36.33% improvement in reconstruction accuracy. This indicates that the calibration precision meets the requirements for high-precision 3D reconstruction.

## Figures and Tables

**Table 1:** The four methods’ average, SE, and maximum errors (unit: pixel).

Method	Average Error		SE		Maximum Error	
	Left	Right	Left	Right	Left	Right
DLT	0.0912	0.0973	0.0128	0.0108	0.172	0.147
Zhang	0.0415	0.0405	0.0052	0.0055	0.0737	0.0759
Calibration toolbox	0.0367	0.0376	0.0048	0.0052	0.0712	0.0709
In this paper	0.0208	0.0219	0.0034	0.0038	0.0308	0.0311

**Table 2:** The time required for the four calibration methods (s).

Method	DLT	Zhang	Calibration Toolbox	Proposed Method
Time	17.94	18.76	20.21	29.74

## Data Availability

Data are contained within the article.

## Abbreviations

Since there are many abbreviations of less commonly used terms in the experimental section of this paper, we have provided a detailed list of abbreviations in the table below:

**Abbreviations**	**Detailed Explanation**
DE	Differential Evolution Algorithm
GA	Genetic Algorithm
PSO	Particle Swarm Optimization
RCCPSO	Radical-Conservative Cooperative Particle Swarm
DLT	Direct Linear Transformation
Zhang	Zhang’s calibration method
